# Comparison of Bacterial Expression Systems Based on Potato Virus Y-like Particles for Vaccine Generation

**DOI:** 10.3390/vaccines10040485

**Published:** 2022-03-22

**Authors:** Anete Ogrina, Dace Skrastina, Ina Balke, Ieva Kalnciema, Juris Jansons, Martin F. Bachmann, Andris Zeltins

**Affiliations:** 1 Plant Virology Laboratory, Latvian Biomedical Research and Study Centre, LV-1067 Riga, Latvia; anete.ogrina@biomed.lu.lv (A.O.); daceskr@biomed.lu.lv (D.S.); inab@biomed.lu.lv (I.B.); ieva.kalnciema@biomed.lu.lv (I.K.); jansons@biomed.lu.lv (J.J.); 2 Department of BioMedical Research, University of Bern, 3008 Bern, Switzerland; martin.bachmann@dbmr.unibe.ch

**Keywords:** virus-like particles, *E. coli*, expression, potato virus Y, Fel d 1

## Abstract

Plant-based virus-like particle (VLP) vaccines have been studied for years, demonstrating their potential as antigen-presenting platforms. In this paper, we describe the development of, and compare between, simple *Escherichia coli*-based antigen display platforms for the generation of potato virus Y (PVY) VLP-derived vaccines, thus allowing the production of vaccines from a single bacterial cell culture. We constructed four systems with the major cat allergen Fel d 1; namely, direct fusion with plant virus PVY coat protein (CP), mosaic PVY VLPs, and two coexpression variants of conjugates (SpyTag/SpyCatcher) allowing coexpression and conjugation directly in *E. coli* cells. For control experiments, we included PVY VLPs chemically coupled with Fel d 1. All constructed PVY–Fel d 1 variants were well expressed and soluble, formed PVY-like filamentous particles, and were recognized by monoclonal Fel d 1 antibodies. Our results indicate that all vaccine variants induced high titers of anti-Fel d 1 antibodies in murine models. Mice that were immunized with the chemically coupled Fel d 1 antigen exhibited the highest antibody titers and antibody–antigen interaction specificity, as detected by binding avidity and recognition of native Fel d 1. IgG1 subclass antibodies were found to be the dominant IgG class against PVY–Fel d 1. PVY CP-derived VLPs represent an efficient platform for the comparison of various antigen presentation systems to help evaluate different vaccine designs.

## 1. Introduction

One of the greatest vaccination challenges is the generation of long-lasting and protective immune responses against various pathogens including bacterial or viral infections, as well as against autoimmune and cancer-related illnesses. Vaccine construction strategies using icosahedral or filamentous plant-based virus-like particles (VLPs) are increasingly harnessed and have been proven to be excellent platforms for antigen presentation [1].

VLPs are self-assembling competent protein structures with identical or highly related overall structures to their corresponding native viruses, representing ideal building blocks to create a variety of new nanomaterials for different purposes, including the active ingredients of different vaccines. One of the most important VLP properties is the ability to mimic viral infection and induce strong immune responses in mammalian organisms, the effects of which are comparable to those induced by native infectious viruses. Plant virus-derived VLPs are viral particles that are noninfectious in mammals, and are very stable, easy to produce, and relatively simply engineered by introducing different antigens at high density on the VLP surface [2], resulting in an effective immune response [3]. Several excellent review articles about VLP immunological properties, including plant virus-derived vaccines, have recently been published [4,5,6,7].

**Figure 1:**
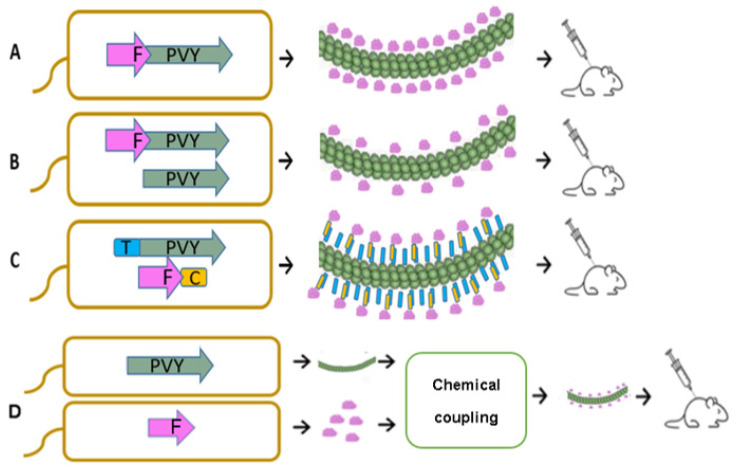
Principal schemes of vaccine production processes using *E.* *coli* expression systems; (**A**)—direct antigen fusion to the coat protein (CP) of filamentous PVY VLPs; (**B**)—coexpression of antigen-containing PVY CP and unmodified PVY CP for the production of mosaic VLPs; (**C**)—coexpression of vaccine components, allowing the formation of VLP–antigen conjugates directly in *E.* *coli* cells. F–Feld1 protein; (**D**)—chemical coupling of PVY VLPs and Fel d 1 protein. PVY–PVY coat protein; T—SpyTag; C—SpyCatcher.

In VLP vaccine construction, several approaches may be considered, including genetic, enzymatic, chemical, and physical processes [1]. In some cases, these methods can be combined. Genetic modifications include techniques that allow the introduction of coding sequences of antigenic peptides or even whole antigens in virus *CP* genes, resulting in N-, C-, or internal fusions of antigens (Figure 1A). When CP fusion with a chosen antigen affects VLP formation due to antigen size or biochemical properties, mosaic VLPs can be constructed to reduce steric hindrance. As shown in a recent paper, mosaic VLPs are formed as a result of a coexpression of antigen-containing and unmodified *CP* genes, leading to VLPs containing antigen fusion and unmodified viral CPs (Figure 1B) [8].

Chemical and enzymatic approaches are broadly used for the introduction of antigens into the structure of carrier VLPs. VLPs and the chosen antigen are expressed and purified separately, and are linked using chemical crosslinking agents or enzymes such as transpeptidase or isopeptidase. Chemical or enzymatic approaches in VLP vaccine construction have several advantages, such as no size or structural limits for the antigen of choice and no influence on the antigen incorporation process on VLP assembly [1]. A recently developed efficient enzymatic system is based on spontaneous isopeptide bond formation between two conjugate partners, SpyTag (13 amino acids, AAs) and SpyCatcher (138 AAs), originating from the *Streptococcus pyogenes* protein CnaB2 [9,10]. This system allows the introduction of chosen antigens into the VLP structure by recombinantly attaching each partner to components of interest [11,12,13]; therefore, this approach may simplify the vaccine production process (Figure 1C) [14].

The main cat allergen from *Feline domesticus*, Fel d 1, is responsible for most allergies caused by cats worldwide, and cat allergies remain a major problem despite several new treatment approaches [15]. Our previous studies have confirmed that Fel d 1 is a very efficient model antigen for vaccine concept representation due to its well described features, such as a high protein production rate and immunogenicity [16,17]. It has previously been tested as a part of a conjugate vaccine using icosahedral plant VLPs derived from a cucumber mosaic virus (CMV) as a VLP carrier, resulting in strong immune responses, including a high-level production of neutralizing antibodies (nAbs) against Fel d 1 in Fel d 1-sensitized mice [16] and cats [17]. In this case, we used the strategy of covalent coupling where the Fel d 1 antigen was chemically conjugated to CMV VLPs, resulting in highly immunogenic particles that induced stronger antibody (Ab) responses than free Fel d 1 or Fel d 1 mixed with VLPs (Figure 1D) [16].

Our previous results using filamentous VLPs, derived from potato virus Y (PVY) as a vaccine carrier for the hepatitis B viral (HBV) preS1 epitope, demonstrated a strong anti-preS1 immune response, even in the absence of adjuvants [18]. Furthermore, PVY VLPs contain several surface exposed lysins that have been used for the successful chemical coupling of milbemycin A3/A4 derivatives [19]. These data suggest the potential use of PVY VLPs as a new antigen-displaying platform. Our recent study revealed that icosahedral plant-based VLPs are more efficient at producing higher immune responses and draining kinetics to secondary lymphoid organs than filaments of the same genetic background [20]. However, considering the results of our previous studies on filamentous PVY VLPs, we decided to test different vaccine designs based on these VLPs.

In this study, we selected Fel d 1 as a model antigen for the comparison of three vaccine construction strategies. We demonstrate that PVY CP-derived VLPs can be engineered for antigen incorporation, resulting in simple *E. coli*-based antigen display platforms, and allowing the production of vaccines from a single bacterial production strain. There are several systems with the major cat allergen Fel d 1, namely; direct fusion with plant virus PVY CPs (Figure 1A), mosaic VLPs (Figure 1B), and two coexpression variants of VLP–antigen conjugates using Spy-Tag and Spy-Catcher systems (Figure 1C) which were constructed and tested in an *E. coli* expression system for VLP formation. The VLPs were characterized and used for immunological studies. In addition, recombinant Fel d 1 (rFel d 1) was chemically coupled to PVY VLPs and used as a control for immunization experiments (Figure 1D). These results can be used to evaluate the most eligible vaccine development process based on filamentous plant VLPs

## 2. Materials and Methods

### 2.1. Cloning of the Fel d 1 Gene with a G4S Linker at the 5′ End of the PVY CP Gene

The recombinant sequence of Fel d 1, which has an additional 15 aa linker sequence of (GGGGS)_3_ genetically fused between two Fel d 1 chains for more flexibility, has been described previously [16]. For further subcloning, we introduced flanking *Nco*I and *Bam*HI sites in the *Fel d 1* gene by PCR mutagenesis using primers Fel_NcoF (5′-ACC ATGGGAAATGACGAAATTTGTCCGGCAGTTAAACGT-3′) and Fel_BamR (5′-GGATCCACGACCCAGGGT ATTCAGTTTCAGA-3′), following the recommended manual for Taq DNA polymerase usage (Thermo Fisher Scientific, Waltham, MA, USA), and adjusting the annealing temperature to 50 °C for 45 s. The corresponding PCR product was then analyzed in a 0.8% agarose gel. After purification with a GeneJet Gel Extraction kit (Thermo Fisher Scientific, USA), the PCR product was ligated into the pTZ57R/T plasmid (Thermo Fisher Scientific, USA), which was later transformed into *E. coli* XL1 Blue cells (Novagen, Madison, WI, USA) for plasmid amplification. Three plasmid clones containing inserts were sequenced using a BigDye cycle sequencing kit (Thermo Fisher Scientific, USA) and an ABI Prism 3100 genetic analyzer (Applied Biosystems, Bedford, MA, USA) for sequence identity. A plasmid clone, pTZ-Fel d 1, without PCR errors was used for further subcloning.

### 2.2. Cloning of the PVY CP–NG4S Direct Fusion and Mosaic System with Fel d 1

The plasmid pET-PVY-CP-NG4S was constructed previously [18] and used for insertion of the Fel d 1 antigen coding sequence. The *Nco*I/*Bam*HI fragment from pTZ-Fel d 1 was subcloned into the same sites of pET-PVY-NG4S, resulting in the expression plasmid pET-PVY-NG4S-Fel d 1 (direct fusion; for plasmid map see Supplementary Figure S1).

For mosaic particle construction, the *PVY-CP* gene was amplified by PCR as previously described, using the primers PVY-NdeF (5′-ACATATGGGAAATGACACAATCGATGCA-3′) and PVY-XhoR (5′-ACTCGAGTTACATGTTCTTCACTCCAAGTAGAGTATGCA-3′).

The corresponding PCR product was then ligated into the pTZ57R/T plasmid (Thermo Fisher Scientific, USA) and transformed into *E. coli* XL1 Blue cells (Novagen, USA). Three plasmid clones containing the insert were sequenced using a BigDye cycle sequencing kit (Thermo Fisher Scientific, USA) and an ABI Prism 3100 genetic analyzer (Applied Biosystems, USA) for identification of the plasmid clone without sequence errors. The obtained PVY *CP* DNA fragment containing the flanking *Nde*I and *Xho*I sites was then ligated into the pETDuet-1 (Novagen, USA) vector cut with the same restriction enzymes. Furthermore, the *Nco*I/*Hind*III fragment from pET-PVY-CP-NG4S was ligated into the pETDuet plasmid, which already contained the PVY–CP gene. The resulting expression vector contained two *PVY CP* genes, the first of which had *Nco*I*/Bam*HI sites for antigen subcloning and a flexible Gly-Ser linker. Finally, the *Fel d 1* gene was introduced into the pETDuet-derived plasmid, resulting in an expression vector for the production of mosaic VLPs (Supplementary Figure S2).

### 2.3. Cloning of Covalently Binding Protein Partners SpyCatcher and SpyTag

The sequence of the *SpyCatcher* gene was obtained from GenBank (JQ478411.1) [9]. The coding DNAs for full-size and truncated variants of the *SpyCatcher* gene were received as a product of commercial gene synthesis. We refer to the two tested SpyCatcher domains as SpyCatcher2 (*SpyC2*; full-size gene) and SpyCatcher3 (*SpyC3*; truncated version) [21].

To obtain both *SpyCatcher* genes with the additional *Nde*I and *Xho*I restriction sites convenient for subcloning, we used PCR mutagenesis as previously described, with the primers SpyC2_NdeF (5′-ACATATGGTTGATACCCTGAGCGGTCT-3′) and SpyC_XhoR (5′-GTAAAGCAACCAAAGGTGATGCACATATTGGATCCGGTACTAGTTAATAAGCTTCTCGAGT-3′) for the *SpyC2* sequence, and SpyC3_NdeF (5′-ACATATGAGCGATAGCGCAACCCACATCAAATTCAG-3′) and SpyC_XhoR for the *SpyC3* sequence. Both corresponding PCR products were then analyzed in a 0.8% agarose gel. After purification with a GeneJet Gel Extraction kit (Thermo Fisher Scientific, USA), PCR products were ligated into the pTZ57R/T plasmid (Thermo Fisher Scientific, USA). After transformation of *E. coli* XL1 Blue cells and plasmid DNA isolation, three plasmid clones from each variant were sequenced using a BigDye cycle sequencing kit (Thermo Fisher Scientific, USA) and an ABI Prism 3100 genetic analyzer (Applied Biosystems, USA) for identification of plasmid clones without PCR errors. Correct clones for *SpyC2* and *SpyC3* were then used for further subcloning into the commercial vector plasmid pRSF-Duet1 (Novagen, USA). Using the introduced restriction sites *Nde*I*/Xho*I*,* corresponding fragments from pTZ-SpyC2 and pTZ-SpyC3 were subcloned into the pET-RSFDuet-1 plasmid, resulting in plasmids pRSF-Duet-SpyC2 and pRSF-Duet-SpyC3.

For construction of Fel d 1 antigen–SpyCatcher fusions, pTZ-Fel d 1 was digested with *Bam*HI*/Xho*I*,* and the corresponding fragment was ligated into pRSF-Duet-SpyC2 and pRSF-Duet-SpyC3.

As previously described, the coding sequence for *SpyTag* (*SpyT*) was introduced into the PVY *CP–NG4S* gene by PCR mutagenesis using the primers pet_BglF (5′-GATCGAGATCTCGATCCCGCGAA-3′) and PVY_SpTBamR (5′-GGATCCTTTGGTCGGTTT ATACGCATCCACCATCACAATATGCGCGTCATTTCCCATGGTATATCTCCTTCT-3′). The corresponding PCR product after purification with a GeneJet Gel Extraction kit (Thermo Fisher Scientific, USA) was ligated into the pTZ57R/T plasmid (Thermo Fisher Scientific, USA). *E. coli* XL1 Blue cells were used for plasmid amplification, resulting in the plasmid pET-PVY-NG4S-SpyT. To avoid PCR errors, three plasmid clones were selected for sequencing and analysis using a BigDye cycle sequencing kit (Thermo Fisher Scientific, USA) and an ABI Prism 3100 genetic analyzer (Applied Biosystems, USA). Furthermore, the *Bgl*II/*Bam*HI fragment from a pTZ plasmid clone containing the *SpyTag* sequence was subcloned into the pET-PVY-CP-NG4S vector, resulting in an expression vector coding for the *PVY* gene containing a coding sequence for SpyTag (SpyT).

To achieve covalent binding directly between the protein partners SpyCatcher and SpyTag in recombinant *E.* *coli*, we subcloned both coding sequences into one expression plasmid using the expression vector pET-PVY-NG4S-SpyT as a vector plasmid. Corresponding *Hind*III*/Blp*I fragments from pRSF-Duet-SpyC2-Fel d 1 and pRSF-Duet-SpyC3-Fel d 1 were subcloned into pET-PVY-NG4S-SpyT, resulting in two expression vectors, pET-PVY-NG4S-SpyT-SpyC2-Fel d 1 (Supplementary Figure S3) and pET-PVY-NG4S-SpyT-SpyC3-Fel d 1, respectively (Supplementary Figure S4).

### 2.4. Expression and Purification of PVY VLPs and PVY Containing Fel d 1 VLPs

*E. coli* C2566 cells (New England Biolabs, Ipswich, MA, USA) were transformed with PVY *CP*-containing plasmids, which were then expressed and purified as previously described [18]. The same transformation was performed for each construct containing the *Fel d 1* antigen. The clones with the highest expression levels of the target protein were selected afterwards, and *E. coli* cultures were grown in a 2TY medium (1.6% tryptone, 1.0% yeast extract, 0.5% NaCl) containing a corresponding antibiotic ((kanamycin (25 mg/L) or ampicillin (50 mg/L)) on a rotary shaker (200 rev/min; Infors, Bottmingen, Switzerland) at 30 °C to an OD600 of 0.8–1.0. Then, 0.2 mM isopropyl β-d-1-thiogalactopyranoside (IPTG) and 5 mM MgCl_2_ were added to induce the cell cultures. Cells were grown on a rotary shaker at 20 °C for 18 h and collected by low-speed centrifugation. The resulting biomass was kept frozen at −70 °C. After thawing on ice, the biomasses containing wild-type (wPVY), mosaic PVY VLPs containing a NG4S-linker (mPVY), direct fusion (PVY-NG4S-Fel d 1), and mosaic VLPs containing the Fel d 1 protein (mPVY-NG4S-Fel d 1), were suspended in a 1× PBS buffer containing 5 mM β-mercaptoethanol (β-ME) and 0.5% TX-100 (buffer A), and were disrupted by ultrasonic treatment. For PVY-NG4S-SpyT/SpyC2-Fel d 1 and PVY-NG4S-SpyT/SpyC3-Fel d 1, the biomass was suspended in a 20 mM TRIS pH 7.0 buffer supplemented with 150 mM NaCl (buffer B) and an additional 5 mM β-ME and 0.5% TX-100, and was disrupted by ultrasonic treatment. Insoluble proteins and cell debris were removed by centrifugation (15,557× *g*, 10 min at 5 °C).

All PVY VLPs were separated from cellular proteins using ultracentrifugation (Optima L—100XP; SW32 rotor, Beckman, USA; at 106,559× *g* (25,000 rpm), 6 h, 18 °C) in a sucrose gradient (20–60% sucrose in buffer A (wPVY, mPVY, PVY-NG4S-Fel d 1, and mPVY-NG4S-Fel d 1)) or in buffer B ((PVY-NG4S-SpyT/SpyC2-Fel d 1 and PVY-NG4S-SpyT/SpyC3-Fel d 1) without β-ME and TX-100). The gradient was divided into six fractions and prepared by carefully filling 6 mL of each solution, one at a time, in descending order (60–20%) with a long needle syringe in tubes (36 mL, for SW—32 rotor, Beckman, Brea, CA, USA), and overlapping the sample on top of all layers. After centrifugation, all fractions were collected in reverse order, starting with the 60% sucrose fraction, and then analyzed by SDS–PAGE. Fractions containing proteins of interest (PVY CP or CP–Fel d 1) were pooled and dialyzed to remove sucrose against 100 volumes of buffer A or B in a 12–14 kDa Spectra/Por 4 dialysis membrane (Spectrum Laboratories, Calgary, Canada). If necessary, the samples were concentrated using an Amicon Ultra-15 100 K filtration unit (Merck–Millipore, St. Louis, MO, USA).

All steps for the expression and purification of VLPs were monitored by SDS–PAGE using 12.5% gels, Western blot (WB), and agarose gel analysis. The concentrations of the proteins were estimated using a Qubit™ fluorometer (Thermo Fisher Scientific, USA) with a Qubit™ Protein Assay Kit (Thermo Fisher Scientific, USA), in accordance with the manufacturer’s recommendations. Concentrated VLP solutions were stored at 4 °C.

### 2.5. Transmission Electron Microscopy (TEM)

The visualization of purified PVY CP-derived VLPs was done with uranyl acetate negative staining. First, 5 µL of the sample (1 mg/mL) was absorbed on carbon formvar-coated 300 Mesh Copper grids (Agar Scientific, Stansted, UK; 2 grids per sample were prepared) and incubated for 3 min. The grids were then washed with 1 mM ethylenediaminetetraacetic acid (EDTA) and negatively stained with 0.5% uranyl acetate aqueous solution. The grids were analyzed with a JEM-1230 electron microscope (JEOL, Tokyo, Japan) at an accelerating voltage of 100 kV and a minimum of five electrograph pictures were made per sample.

### 2.6. Chemical Coupling of PVY CP VLPs and Fel d 1

rFel d 1 was purified using a His-tag column as described previously [16]. The purified rFel d 1 was conjugated to PVY CP VLPs using the crosslinker succinimidyl-6-(β-maleimidopropionamido) hexanoate (SMPH; Thermo Fisher Scientific, USA). A 5-fold molar excess of SMPH to PVY VLPs was used for the reaction at 23 °C for 1 h. Unreacted SMPH was removed by an Amicon Ultra-15 50 K centrifugal filter (Merck–Millipore, USA); VLPs were further washed with 1× PBS four times (4 × 6 min) at 3214× *g* (5000 rpm) and 5 °C. The antigen prior to chemical conjugation was treated with a 10-fold molar excess of mild reducing agent tris (2-carboxyethyl) phosphine (TCEP; Sigma–Aldrich, Burlington, MA, USA) for 10 min at room temperature (RT). The coupling was performed by adding a 4-fold molar excess of rFel d 1 to the SMPH-derivatized PVY VLPs at 23 °C for 3 h by shaking at 1400 rpm/min on a DSG Titertek (Flow Laboratories, Oldham, UK). Unbound rFel d 1 was removed using an Amicon Ultra–15 100 K centrifugal filter (Merck–Millipore, USA). All stages of coupling were analyzed by SDS–PAGE and the integrity of VLPs was confirmed by TEM.

### 2.7. Immunological Studies

#### 2.7.1. Western Blot (WB) Analysis

For WB analysis, protein samples were separated by SDS–PAGE and transferred to an Amersham Protran 0.45 µm nitrocellulose membrane (GE Healthcare, Piscataway, NJ, USA) using a semidry apparatus with parameters of 250 V, 45 A, and 45 min. After blocking the membrane in a PBS solution containing a 1% alkali–soluble casein (Merck–Millipore, USA), the membrane was incubated overnight (ON) at 4 °C in anti-PVY or anti-Fel d 1 Ab-containing solutions (diluted at 1:1000 in PBS with 1% alkali–soluble casein) obtained from mice that were immunized with PVY CP VLPs [18] or a recombinant antigen (rFel d 1) [16]. The membrane was washed with a TBS buffer (150 mM NaCl; 10 mM Tris pH 7.5) for 15 min and then incubated at RT for 3 h with horseradish peroxidase-conjugated anti-mouse IgG (Sigma–Aldrich, USA) that was diluted at 1:1000 in PBS, supplemented with 1% alkali–soluble casein. The membrane was washed with TBS for 15 min two times. The signal bands were developed by incubating the membrane in a TBS buffer supplemented with peroxidase substrates (0.002% *o*-dianisidine and 0.03% hydrogen peroxide).

#### 2.7.2. Mouse Vaccination

For immunogenicity testing, 6–8 weeks old female BALB/c mice (5 per group) were purchased from Laboratory Animal Centre, University of Tartu (Estonia). A volume of 50 µg of each VLP (direct, mosaic, SpyT/SpyC2, SpyT/SpyC3, and chemically coupled) were diluted in 300 µL of sterile PBS and used for subcutaneous injection in mice without adjuvant on Day 0. Mice received a similar booster dosage on Days 14 and 28. One 100 µL serum sample from each mouse was collected each week on Days 7, 14, 28 and 42. The experimental protocol was approved by the Animal Protection Ethical Committee of the Latvian Food and Veterinary Service (permission no. 89).

#### 2.7.3. The Enzyme-Linked Immunosorbent Assay (ELISA)

For total IgG Ab titer determination against Fel d 1 and PVY CP in sera of immunized mice, purified samples of rFel d 1 and PVY VLPs in a concentration of 10 µg/mL in 50 mM sodium carbonate buffer (pH 9.6, 100 µL per well) were coated on 96-well ELISA plates (Nunc Immuno MaxiSorp, Thermo Fisher Scientific, Rochester, NY, USA), and stored at 4 °C ON. The next day, blocking with 1% BSA in PBS at 37 °C for 1 h was performed, and serially diluted mouse sera were added to the plates and incubated at 37 °C for 1 h. Afterwards, plates were washed three times with PBS containing 0.05% Tween-20. A volume of 100 µL of horseradish peroxidase, labelled anti-mouse Ab (Sigma–Aldrich, USA), was added at a 1:10,000 dilution per well. After incubation at 37 °C for 1 h, plates were washed again, and the *o*-phenylenediamine dihydrochloride (OPD) (Sigma–Aldrich, USA) substrate was added for color development. Optical absorbance was measured with a Labsystems 352 Multiscan MS microplate reader (Sweden) at 492 nm. The endpoint titers were calculated at the highest serum dilution, resulting in an absorbance value exceeding three-fold that of the negative control (serum obtained from nonimmunized mice).

For monoclonal Fel d 1 Ab ELISA tests, two sets of plates were coated with 10 µg/mL of each variant PVY–Fel d 1 VLPs at 4 °C ON. After blocking with 1% BSA in PBS at 37 °C for 1 h, serial dilutions of two types of Fel d 1 monoclonal Abs (mAb) against chain 1 (MA-3E4 and MA-6F9; Indoor Biotechnologies, Cardiff, UK) were added to the plates and incubated at 37 °C for 1 h, and then washed three times with PBS containing 0.05% Tween-20. The rest of the procedure was identical to that described above.

For the detection of IgG1 and IgG2a subclasses, isotype-specific ELISA was performed for the detection of anti-PVY and anti-Fel d 1 Ab, using mouse mAb isotyping reagent ISO2 (Sigma–Aldrich, USA) as secondary Abs, using the peroxidase conjugate of monoclonal anti-goat/sheep IgG Abs (Sigma–Aldrich, USA). The endpoint titers were calculated as stated above.

#### 2.7.4. Avidity ELISA

To determine the avidity of IgG Abs, two sets of plates were prepared. Both were coated with 10 µg/mL PVY CP VLPs and Fel d 1-C6H-CG. After serum incubation, one set of plates was washed three times for 5 min with a 50 µL/well of a solution containing 7 M urea in PBS supplemented with 0.05% Tween-20. The other set was washed with the same amount of PBS with 0.05% Tween-20. Between washing steps, all the plates were washed with PBS, with 0.01% Tween-20 as usual. The rest of the procedure was identical to that described above.

#### 2.7.5. ELISA for Native Fel d 1

To test whether the Abs induced by PVY–Fel d 1 VLP variants can recognize the native Fel d 1 (nFel d 1) protein, a sample of nFel d 1 (Indoor Biotechnology, UK) in a concentration of 10 µg/mL in a 50 mM sodium carbonate buffer (pH 9.6, 100 µL per well), was used to coat 96-well ELISA plates (Nunc Immuno MaxiSorp, Thermo Fisher Scientific, Rochester, NY, USA) and stored at 4 °C ON. To further the blocking, 1% BSA in PBS at 37 °C for 1 h was performed, and serially diluted mouse sera (5 µL from each sample) were added to the plates and incubated at 37 °C for 1 h. Afterwards, the plates were washed three times with PBS containing 0.05% Tween-20. A volume of 100 µL of horseradish peroxidase labelled anti-mouse Ab (Sigma–Aldrich, USA) was added at a 1:10,000 dilution per well. After incubation at 37 °C for 1 h, plates were washed again, and the *o*-phenylenediamine dihydrochloride (OPD) (Sigma–Aldrich, USA) substrate was added for color development. Optical absorbance was measured with a Labsystems 352 Multiscan MS microplate reader (Sweden) at 492 nm. The endpoint titers were calculated as the highest serum dilution that resulted in an absorbance value exceeding three-fold that of the negative control (serum obtained from nonimmunized mice).

## 3. Results and Discussion

### 3.1. Construction and Characterization of PVY CP VLPs Containing Fel d 1

Our previous studies revealed that some filamentous plant VLPs (PVY, potato virus M (PVM)) can accommodate comparably large protein domains on their surface as N– or C–terminal fusions without influencing VLP self-assembly [18,22]. First, we constructed direct fusions from a previously described PVY *CP* sequence with an introduced Gly-Ser linker [18] between *CP* and antigen sequences to reduce the influence of foreign sequences, which may interfere with VLP formation, further purification, and identification. The *Fel d 1* coding sequence was introduced in the N–terminal part of PVY–NG4S-CP, and expressed directly in *E. coli* cells (see plasmid map in Supplementary Figure S1).

**Figure 2:**
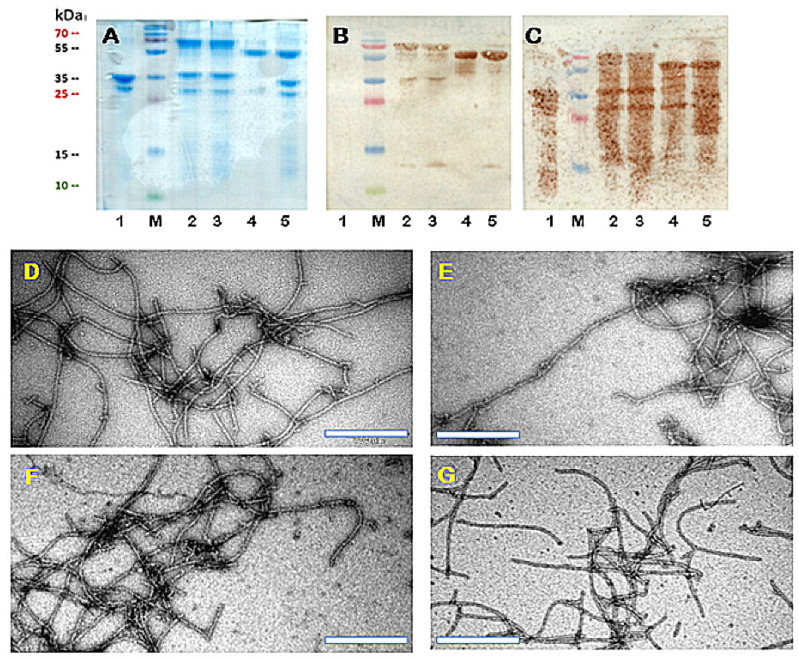
Characterization of PVY CP VLPs with the Fel d 1 antigen. (**A**) Coomassie-stained SDS–PAGE gel of PVY–Fel d 1 vaccine variants at a concentration of 2 mg/mL; (**B**) the same samples as in (**A**) analyzed by Western blot using anti-Fel d 1 antibodies; (**C**) the same samples as (**A**) analyzed by Western blot using anti-PVY antibodies. Electron micrographs of negatively stained VLPs; (**D**) direct fusion of PVY-NG4S-Fel d 1; (**E**) mosaic PVY-NG4S-Fel d 1; (**F**) PVY-SpyT/SpyC2-Fel d 1; (**G**) PVY-SpyT/SpyC3-Fel d 1, scale bars 500 nm. M—PageRulerTM Plus Prestained Protein Ladder, 10 to 250 kDa (Thermo Fisher Scientific, USA), 1—PVY-NG4S-PVY (mosaic; control); 2—PVY-SpyT-SpyC2-Fel d 1; 3—PVY-SpyT-SpyC3-Fel d 1; 4—PVY-NG4S-Fel d 1; 5—PVY-NG4S-Fel d 1 PVY-(mosaic).

To construct the mosaic system, we incorporated the wPVY *CP* and PVY *CP-NG4S-Fel d 1* genes into the expression vector pET-Duet1, allowing the formation of mosaic particles (mPVY-NG4S-Fel d 1) after expression in *E. coli*. The protein size of PVY-NG4S-Fel d 1 is ~55 kDa (Figure 2A; track 4). For mosaic VLPs, we identified two protein bands—wPVY with a size of ~35 kDa and PVY-NG4S-Fel d 1 with a size of ~55 kDa—after SDS–PAGE analysis, as expected (Figure 2A; track 5). The TEM results confirmed that the expression of both systems resulted in similar filamentous VLPs with an average size of 400–800 nm (Figure 2D,E), similar to what was previously described [18], suggesting that VLPs can tolerate Fel d 1 insertion without significantly interfering with their structure. Assuming that direct fusion leads to 100% incorporation of the Fel d 1 antigen, densitometric analysis of SDS–PAGE gels revealed approximately 63% Fel d 1 incorporation in the mosaic particles. The output of VLPs varied between 11 and 12 mg/g biomass.

Several previously developed SpyTag/SpyCatcher-based platforms involved individually purified components which were then linked together in a separate process [10,23,24]. However, there are examples of the reaction between SpyTag- and SpyCatcher-containing proteins forming covalent bonds directly in the cells of recombinant bacteria or even in plants [23,25].

**Figure 3:**
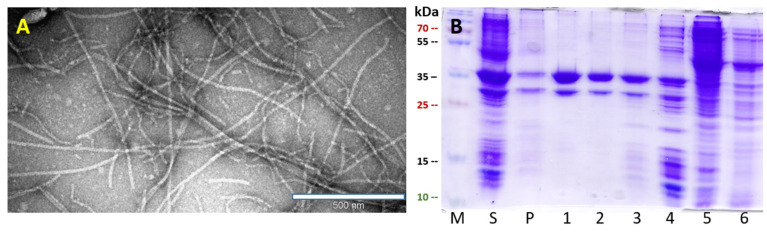
PVY-NG4S-SpyT analysis. (**A**) Electron micrographs of negatively stained VLPs; (**B**) Coomassie-stained SDS–PAGE. M—PageRulerTM Plus Prestained Protein Ladder (Thermo Fisher Scientific). S—soluble proteins in cell lysate; P—insoluble proteins in cell lysate; 1–6—sucrose gradient fractions (60–0% sucrose).

To test whether SpyTag-containing PVY VLPs can directly form a conjugate with a SpyCatcher–Fel d 1 protein in the same bacterial cell, we first genetically introduced PVY *CP-NG4S* with a *SpyTag* sequence using PCR mutagenesis and expressed it in *E. coli* strain C2566. The majority of the protein was soluble and well expressed, with a size of ~35 kDa (Figure 3B). TEM also confirmed PVY-like VLP formation in various sizes ranging from 200–1000 nm (Figure 3A). In a second step, two SpyCatcher peptide sequences (full size *SpyC2* and N–terminally truncated *SpyC3*) [14] were genetically fused with *Fel d 1* and then used for further subcloning. To introduce *SpyTag/SpyCatcher* partner sequences into one expression system, we used the commercial vector plasmid pRSF-Duet, which has two *T7* promoters and multiple cloning sites, one for each genetically fused *PVY-NG4S-SpyTag/SpyCatcher-Fel d 1* partner.

Both expression vectors pRSFDu-PVY-NG4S-SpyT/SpyC2-Fel d 1 and pRSFDu-PVY-NG4S-SpyT/SpyC3-Fel d 1 were used for *E. coli* C2566 transformation. Cloned genes were expressed, and the corresponding VLPs after purification were analyzed using SDS–PAGE gels. The analysis confirmed that SpyTag- and SpyCatcher-derived proteins form conjugates directly in *E. coli* cells. The expected size of conjugated PVY-SpyT/SpyC proteins containing the Fel d 1 antigen was ~70 kDa (Figure 2A; track 2, 3), while the band ~35 kDa corresponds to overlapping antigen-free PVY-NG4S-SpyT (32.6 kDa) and free SpyC2–Fel d 1 (31.3 kDa) or SpyC3–Fel d 1 (29.2 kDa; see Figure 2 and plasmid maps in Supplementary File). The output of VLPs was approximately 10 mg/g biomass.

The morphology of filamentous VLPs was confirmed by TEM (Figure 2F,G), revealing shorter VLP particle formation with sizes ranging from 200–400 nm. Additionally, densitometric analysis revealed approximately 65% Fel d 1 incorporation for PVY-NG4S-SpyT/SpyC2-Fel d 1 and 71% for PVY-NG4S-SpyT/SpyC3-Fel d 1. These results demonstrate that both SpyCatcher versions (full size and truncated) have similar conjugation efficiency, as observed in previous studies [14].

**Figure 4:**
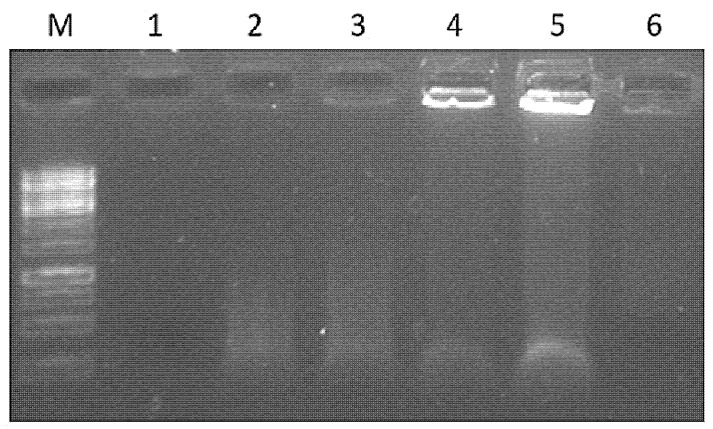
Agarose gel analysis of PVY–Fel d 1 vaccine variants at a concentration of 1.5 mg/mL. M—GeneRuler 1 kb DNA ladder (Thermo Fisher Scientific, USA); 1—wPVY (wild-type; control); 2—PVY-NG4S-Fel d 1 (direct fusion); 3—PVY-NG4S-Fel d 1-PVY (mosaic fusion); 4—PVY-SpyT-SpyC2-Fel d 1; 5—PVY-SpyT-SpyC3-Fel d 1; 6—cPVY–Fel d 1 (chemically coupled). A 0.8% native agarose gel stained with ethidium bromide was used for analysis.

Native filamentous plant viruses contain a comparably low proportion of encapsidated nucleic acids. We previously observed a low nucleic acid content for filamentous VLPs derived from plant viruses [22]. Considering the importance of bacterial nucleic acids in stimulating the immune response, we attempted to identify DNA/RNA in purified VLP samples. Native agarose gel (NAG) analysis of VLP variants after ethidium bromide staining suggested that only four vaccine variants contained visible signals of nucleic acids (Figure 4). Packaged RNA was hardly noticeable for wPVY and cPVY–Fel d 1 VLPs. In contrast, PVY-NG4S-SpyT/SpyC2-Fel d 1 and PVY-NG4S-SpyT/SpyC3-Fel d 1 VLPs contained clearly detectable nucleic acid signals. As shown in Figure 4, a large portion of filamentous VLPs cannot enter the agarose gel due to their large size, and therefore, they appear as bands in NAG pockets.

To further characterize vaccine candidates, we performed WB analysis using an Ab against both Fel d 1 and carrier PVY VLPs. Mosaic VLPs consisting of NG4S-containing and unmodified PVY CPs were used as a control for both analyses. As expected after WB analysis, mPVY did not react with anti-Fel d 1 Abs (Figure 2B). In contrast, all four Fel d 1-containing vaccine prototypes revealed the presence of Fel d 1 in WB analysis by reacting with anti-Fel d 1 Abs (Figure 2B). The WB signals overlapped with the corresponding bands identified in SDS–PAGE: ~70 kDa for PVY–Fel d 1 conjugates and ~35 kDa for free Fel d 1. We also observed a slight degradation of the Fel d 1 protein, resulting in an ~13 kDa degradation product in samples containing both SpyCatcher variants and mosaic VLPs. WB analysis using anti-PVY Abs demonstrated proteolytic degradation of the VLP carrier (Figure 2C). We also noted similar results in our previous experiments [18], where the formation of truncated PVY CP derivatives appeared, possibly due to proteolytic processing by *E. coli* trypsin-like enzymes.

### 3.2. Chemical Coupling of PVY CP with Fel d 1

**Figure 5:**
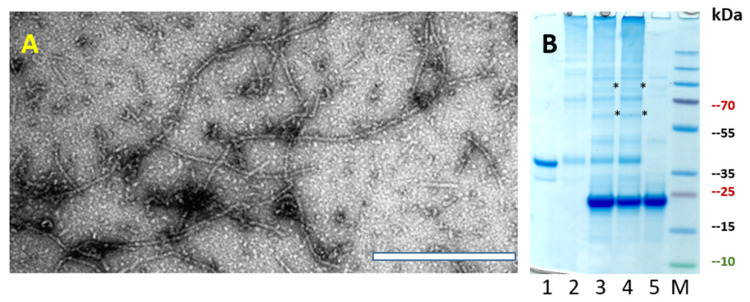
Chemically coupled PVY–Fel d 1 analysis. (**A**) Electron micrographs of negatively stained cPVY–Fel d 1, scale bar 500 nm; (**B**) Coomassie-stained SDS–PAGE gel. 1—PVY; 2—PVY after 5× SMPH derivatization and unreacted SMPH removal; 3—PVY coupling with 4 × Fel d 1-C6H-CG; 4—PVY + 4 × Fel d 1-C6H-CG after partial Fel d 1-C6H-CG removal; 5—rFel d 1-C6H-CG treated with 10× TCEP; M—PageRuler™ Plus Prestained Protein Ladder (Thermo Fisher Scientific); *—coupling product of PVY-rFel d 1.

For the chemically coupled PVY–Fel d 1 (cPVY–Fel d 1) variant, the two principal components, the PVY CP and the antigen, were expressed separately. First, we purified unmodified PVY VLPs using PEG8000/NaCl precipitation and ultracentrifugation in a sucrose gradient, as previously described [18]. Fractions containing PVY CPs were purified, as described in Materials and Methods, for further use. The concentration of PVY CP VLPs was measured using a Qubit™ Protein Assay Kit (Thermo Fisher Scientific, USA), and the samples were analyzed by SDS–PAGE (Figure 5B), confirming the correct size of ~35 kDa. The sample was then mixed with 5× SMPH for derivatization and incubated for 1 h. Unreacted SMPH was removed by buffer exchange on Amicon-Ultra-15 50 K filtration units (Merck–Millipore, USA).

The rFel d 1 protein with a His-tag and C–terminal Cys residue was purified, as described previously, using a Protino Ni-TED 1000 Packed Column (Macherey-Nagel, Bethlehem, PA, USA) [16]. Then, fractions containing the rFel d 1 protein were dialyzed against 1x PBS. Before coupling, rFel d 1 was treated with the reducing agent TCEP, and then, a 4-fold molar excess of rFel d 1 was added to the derivatized PVY–VLP at 23 °C for 2 h. Uncoupled rFel d 1 was removed in Amicon-Ultra-15 100 K filtration units by centrifugation. All stages of chemical coupling were monitored by SDS–PAGE (Figure 5B). Densitometric analysis suggested rFel d 1 incorporation of approximately 24% when calculated from the signal intensities of the unmodified PVY CP and coupling products (Figure 5B; track 4).

Electron microscopy analysis of the final product demonstrated that the PVY-like particles preserved the filamentous VLP structure after the coupling procedure (Figure 5A), although we noticed the presence of much shorter filaments with sizes ranging approximately 100–200 nm. A similar observation about chemical coupling efficiency in rod-shaped nanoparticles was made by Rioux et al. and Therien et al. when analyzing Papaya mosaic virus (PapMV) particle formation after two influenza epitopes (HA11; 9 AA) [26] and (M2e; 26 AA) [27] were coupled to PapPMV CP. TEM revealed that chemical coupling slightly interfered with longer particle formation compared with PapMV WT, suggesting that successful coupling relies on several aspects, such as the size of the chosen epitope, chemical linker, or nanoparticle surface morphology [26,27].

### 3.3. Immunological Characterization of the PVY–Fel d 1 Vaccine Candidates

To characterize the immune stimulation potency of the constructed variants, we performed ELISA to test the antigenicity of VLP-introduced antigens using two commercially available monoclonal Fel d 1 Abs before murine experiments. To elucidate which of the constructed PVY–Fel d 1 variants is the most promising candidate for possible vaccine design, we performed experiments in mice to measure the amount of antigen-specific total IgG as well as IgG subclasses by ELISA. For additional analysis of IgG specificity, avidity ELISAs were performed using sera collected on Day 42. To test whether collected sera from immunized mice were able to recognize a nFel d 1 allergen, we included an ELISA test for total specific IgG Abs.

#### 3.3.1. Monoclonal Antibody ELISA

**Figure 6:**
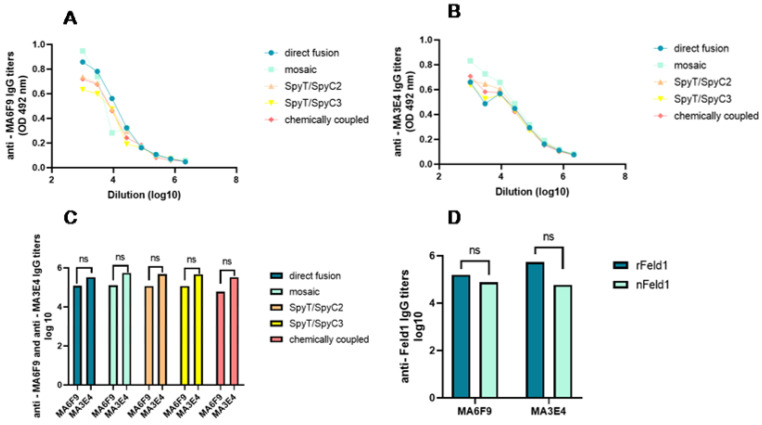
Monoclonal Fel d 1 antibody reaction with displayed Fel d 1 on the surface of vaccine variants. Plates were coated with 10 µg/mL of each VLP. (**A**)—total IgG titers against Fel d 1 monoclonal antibody MA-6F9 (Indoor Biotechnology, USA); (**B**)—total IgG titers against Feld1 monoclonal antibody MA-3E4 (Indoor Biotechnology, USA); (**C**)—comparison of MA-6F9 and MA-3E4 titers of total IgGs against; (**D**)—comparison of monoclonal antibody MA-6F9 and MA-3E4 titers against recombinant Fel d 1 (rFel d 1) or native Fel d 1 (nFel d 1). (**C**,**D**)—Statistical analysis using Student’s *t* test in GraphPad Prism 9.0. Vaccine groups *n* = 5. One representative experiment was performed. A value of *p* > 0.05 was considered statistically significant (ns—not significant).

Before starting in vivo murine experiments, we wanted to characterize the antigenicity of the constructed PVY–Fel d 1 vaccine candidate by measuring recognition with two commercially available Fel d 1 mAbs with ELISA. The nFel d 1 protein consists of two chains (chain-1 and chain-2) [28]; the two chosen commercially available mAbs bind to two different epitopes on the cat *F. domesticus* allergen Fel d 1, both on chain-1 (MA-6F9; MA-3E4; Indoor biotechnologies, UK). For optimal induction of nAbs, it is important that the Fel d 1 incorporated into PVY VLPs is structurally identical to nFel d 1 and is capable of inducing Abs that recognize nFel d 1; therefore, we wanted to establish whether there is a difference in mAb binding to rFel d 1 or nFel d 1. Our results indicated that nFeld1 is equally recognized by both mAbs (Figure 6D) (corresponding to reciprocal titers between 55,000 and 75,000). Surprisingly, MA-3E4 mAbs demonstrated an even higher interaction with the rFel d 1 produced by us (corresponding reciprocal titers for MA-3E4 1:548,000; for MA-6F9 1:158,000).

Similar analysis using these two mAbs was performed for the constructed VLP variants and revealed that both mAbs strongly bound to all PVY–Fel d 1 vaccine variants (Figure 6A,B); MA–3E4 mAbs demonstrated the highest binding to each VLP variant. Although MA–6F9 did not show significant differences in Ab binding to PVY–Fel d 1 vaccine variants, titers against MA–3E4 mAbs were highest for mPVY-NG4S-Fel d 1 (Figure 6C) (corresponding reciprocal titers for MA–6F9 1:130,000; for MA–3E4 1:560,000), indicating that Fel d 1 is well exposed on the surface of these VLPs. PVY-NG4S-SpyT/SpyC2-Fel d 1 and PVY-NG4S-SpyT/SpyC3-Fel d 1 were also capable of binding to MA-3E4 very efficiently (corresponding reciprocal titers for SpyT/SpyC2 1:485,000; for SpyT/SpyC3 1:468 000). Interestingly, the chemically coupled variant produced slightly lower titers for MA–3E4 than other PVY–Fel d 1 vaccine variants (corresponding reciprocal titer 1:330,000). 

#### 3.3.2. Total Levels of IgG

Subcutaneous immunizations of 6 to 8-weeks old female BALB/c mice were performed on Day 0 with 50 µg of the five PVY–Fel d 1 vaccine candidates PVY-NG4S-Fel d 1, mPVY-NG4S-Fel d 1, PVY-NG4S-SpyT/SpyC2-Fel d 1, PVY-NG4S-SpyT/SpyC3-Fel d 1 and cPVY–Fel d 1 without additional adjuvants. Booster doses were given on Days 14 and 28. Total specific IgG immune responses were detected in sera collected on Days 7, 14, and 28, and a final blood collection was performed on Day 42 for ELISA test analysis. Total IgG levels were measured against the levels of carrier PVY CP as well as rFel d 1 and nFel d 1.

**Figure 7:**
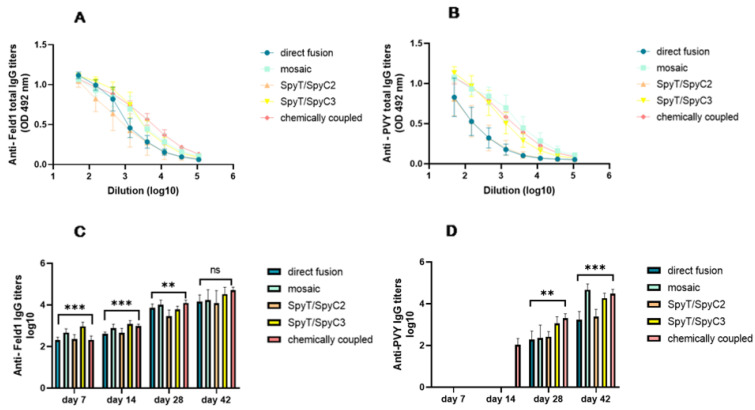
Fel d 1 IgG titer analysis after vaccination with PVY–Fel d 1 vaccine variants. (**A**)—Fel d 1-specific IgG titers on Day 42 for the groups vaccinated with PVY–Fel d 1 variants measured at OD 492 nm; (**B**)—PVY CP-specific IgG titers on Day 42 for the groups vaccinated with PVY–Fel d 1 variants measured at OD 492 nm; (**C**)—Log_10_ values (mean ± SEM) of Feld1-specific IgG titers for the groups vaccinated with PVY–Fel d 1 VLPs on Days 7, 14, 28 and 42; (**D**)—Log_10_ values (mean ± SEM) of PVY CP-specific IgG for the groups vaccinated with PVY–Fel d 1 VLPs on Days 7, 14, 28 and 42. (**C**,**D**)—Statistical analysis using one-way ANOVA in GraphPad Prism 9.0. Vaccine groups *n* = 5. One representative experiment is shown. A value of *p* > 0.05 was considered statistically significant (ns—not significant; ** *p* < 0.001; *** *p* < 0.0001).

Fel d 1-specific Abs were detected very early after the first dose. IgG titers at Day 7 were highest for PVY-SpyT/SpyC3-Fel d 1 (Figure 7C). The same was observed on Day 14. IgG responses against Fel d 1 were enhanced dramatically on Day 28 after the first boost, which led to IgG levels that showed a minimum of a 5.6-fold increase compared with those detected on Day 14, showing that the highest anti-Fel d 1 titers from Day 28 were obtained from mice injected with cPVY–Fel d 1 (corresponding reciprocal titer 1:11,500). 

Each of the IgG response levels against PVY CP and Fel d 1 reached the maximum on the final day (42) after the second boost, demonstrating the highest anti-Fel d 1 titers for cPVY–Fel d 1 (Figure 7A,C) (corresponding reciprocal titer 1:52,938), followed by PVY-SpyT/SpyC3-Fel d 1 (corresponding reciprocal titer 1:36,500). Furthermore, the IgG levels for these two VLPs increased by approximately five-fold after the second boost compared to previously obtained titers, indicating the possibility of a high repetitiveness of antigens on the VLP surface, which has previously been reported to induce high IgG titers [29]. PVY CP-specific Abs were only detectable from Day 28 and were highest against cPVY–Fel d 1 as well as for mPVY-NG4S-Fel d 1 (Figure 7B,D). The obtained results only partly correlate with the data from densitometric analysis of the incorporated Fel d 1 antigen. The immune responses were higher for PVY-SpyT/SpyC3-Fel d 1 (71%) than for PVY-SpyT/SpyC2-Fel d 1 (65%) or mPVY–Fel d 1 (63%), but this could not be considered the main factor because for cPVY–Fel d 1, which had the highest Ab levels, densitometric analysis suggested only 24% Fel d 1 incorporation. Additionally, direct fusion of PVY–Fel d 1 with 100% incorporated Fel d 1 antigen did not result in the highest Ab levels, suggesting that the process of generating an immune response is also influenced by other factors, such as the particle size and RNA content.

To further characterize induced Abs against Fel d 1, we performed an avidity ELISA experiment using sera from Day 42. According to several authors, avidity may be defined as the ability of a polyvalent molecule to form multiple connections of the same kind with ligands tethered to the same surface, or in other words, it characterizes the potential multivalent interaction of an Ab with an antigen [30,31]. Furthermore, polyvalent molecules such as Abs are restricted by the geometry of interaction, meaning that recognition of the surface-bound ligands is linked to topological properties of the surface [31]; therefore, avidity could possibly define the strength and/or number of interactions that determine the quality of produced Abs. As shown for the malaria vaccine, RTS, S, and Ab avidity are a very important factors, which correlates with vaccine efficacy [32].

**Figure 8:**
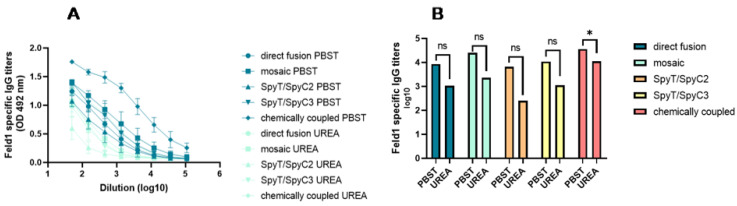
Avidity antibody detection after vaccination with PVY–Fel d 1 variants. (**A**)—Fel d 1-specific IgG titers for the groups vaccinated with PVY–Fel d 1 variants on Day 42 measured at OD_492_. After serum incubation, one plate was treated with PBS with 0.05% Tween 80, and the other plate was treated with 7 M urea in PBS with 0.05% Tween 80; (**B**)—Log_10_ values (mean ± SEM) of Fel d 1-specific IgG titers (shown in a) for the group vaccinated with PVY–Fel d 1 vaccine variants. Statistical analysis was performed using Student’s *t* test in GraphPad Prism 9.0. Vaccine groups *n* = 5. One representative experiment was performed. A value of *p* > 0.05 was considered statistically significant (ns—not significant; * *p* < 0.01).

For avidity tests, most studies rely on ELISA format analysis with different concentrations of chaotropic agents to determine the stability of the antigen–Ab complex. We compared the total IgG levels of two identical plates, where one was washed with 7 M urea while the other was treated as usual. The results indicated that the cPVY–Fel d 1 variant induces Abs with the highest avidity index (AI) values (Figure 8A,B), meaning that 36% of all IgGs were specific and could not be washed away with 7 M urea. PVY–Fel d 1, mPVY–Fel d 1 and PVY-SpyT/SpyC3-Fel d 1 induced Abs with ~9–12% specificity, whereas variants with the least specific Abs included PVY-SpyT/SpyC2-Fel d 1, which resulted in only ~4% specificity. These findings clearly demonstrate that the method of antigen incorporation in VLPs can influence the structure of the Fel d 1 antigen on the VLP surface and result in the generation of Abs with different specificities. 

#### 3.3.3. Subclass Specific Antibody Production

Previously published papers have suggested that several factors, such as the structure and quantity of antigen, as well as the possible route and time period of antigenic stimulation, can affect which IgG subclass of Abs will be produced [33]. The murine IgG family is divided into four major subclasses, namely; IgG1, IgG2a/c, IgG2b and IgG3, elucidating that in particular, the IgG2a subclass is most capable of protecting individuals against an infectious bacterial challenge [34]. It is known that in the absence of nucleic acids that serve as toll-like receptor (TLR) stimulators, nanoparticles such as VLPs induce IgG1-dominated immune responses in mice, whereas the dominating subclass in the presence of nucleic acids becomes a much more protective IgG2a subclass [30]. Furthermore, it has been shown that these two IgG subclasses (IgG2a and IgG1) in mice can define whether the T helper 1 (T_h_1) or T helper (T_h_2) signaling pathway is dominating, respectively [35]. For protective immunity against infection, T_h_1 cells stimulate Ab production toward the IgG2a subclass and are considered the most potent Ab response against most viral and bacterial pathogens [29]. 

Although it is known that viral infections with live or inactivated viruses are mostly restricted to the IgG2a isotype, several papers have confirmed that immunization with many viral proteins or peptides, often combined with adjuvants, leads to an IgG1 Ab response, especially in the absence of naturally packed nucleic acids [36,37,38,39].

**Figure 9:**
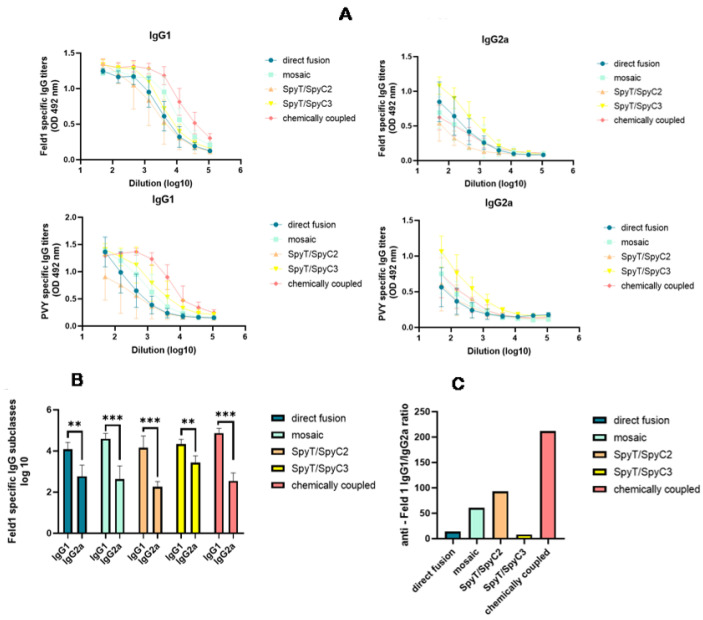
PVY–Fel d 1 variants induce subclass switching by ELISA analysis. (**A**)—Anti–Fel d 1- and anti-PVY CP-specific IgG1 and IgG2a titers measured in Day 42 mouse sera at OD492 nm. ELISA plates were coated with PVY–Fel d 1 variants to detect IgG subclasses in mice vaccinated with PVY–Fel d 1 variants. (**B**)—Log_10_ values of Feld1-specific IgG1 and IgG2a titers measured in Day 42 mouse sera; (**C**)—anti-Fel d 1 IgG1/IgG2a ratio measured from titers used in the experiment shown in (**B**); (**A**,**B**) Statistical analysis using Student’s *t* test in GraphPad Prism 9.0. Vaccine groups *n* = 5. One representative experiment is shown. A value of *p* > 0.05 was considered statistically significant (** *p* < 0.001; *** *p* <0.0001).

Analysis of the induced anti-Fel d 1 Abs revealed an excess of IgG1 over IgG2a in all cases. The highest IgG1 Ab response was produced by mice that were vaccinated with cPVY–Fel d 1 (corresponding reciprocal titer 1:91,000), followed by mPVY–Fel d 1 (corresponding reciprocal titer 1:36,000), whereas PVY-NG4s-SpyT/SpyC3-Fel d 1 produced the highest IgG2a titer (Figure 9A,B) (corresponding reciprocal titer 1:3200) among the groups.

Our previous research on filamentous virus PVM VLPs revealed very low amounts of packaged nucleic acids [22]. We obtained similar results in this study, indicating low amounts of nucleic acids within PVY (Figure 4), which may be a reason for the dominance of IgG1 in mice vaccinated with most of the VLP variants. The lowest IgG1/IgG2a ratio value was obtained in sera from mice immunized with PVY-NG4s-SpyT/SpyC3-Fel d 1 (Figure 9C). These results correlate with the nucleic acid content found in VLPs after NAG analysis. However, this effect was not observed in PVY-NG4s-SpyT/SpyC2-Fel d 1-immunized mice.

#### 3.3.4. Native Fel d 1 Recognition

Sensitization through environmental exposure to the main cat allergen, Fel d 1, continues to be one of the main allergy-related problems worldwide [40]. Moreover, our previously obtained data using the main cat allergen Fel d 1 have confirmed it to be a well characterized, easily produced, and efficient model antigen for vaccine design [16]. To evaluate possible vaccine efficiency between constructed variants in vivo, we tested how well-produced sera from vaccinated mice could recognize the commercially available nFel d 1 antigen isolated from cats (Indoor Biotechnologies, USA).

**Figure 10:**
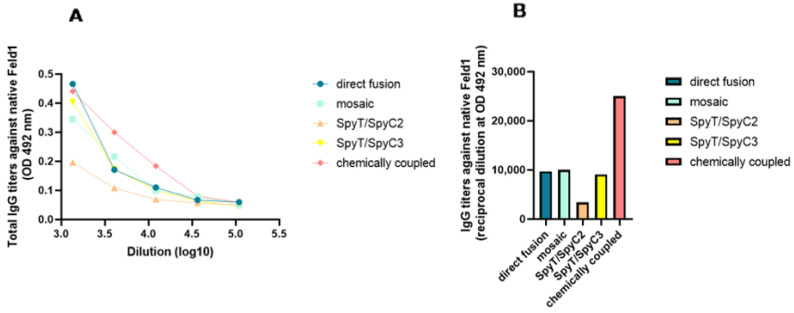
PVY–Fel d 1 variants induced antibodies against Fel d 1 ELISA analysis using native Feld1. (**A**,**B**)—Fel d 1-specific IgG titers against native Feld1 for the groups vaccinated with PVY–Fel d 1 vaccine variants on Day 42 measured at OD_492_. One representative experiment was performed.

After serum incubation on plates with nFel d 1, and all the additional steps as previously described, ELISA was performed. The results were very similar to those obtained with rFel d 1—the highest absorbance values were for cPVY–Fel d 1 after chemical coupling (Figure 10A,B) (corresponding reciprocal titer 1:25,000), which produced a minimum two-fold excess compared to that of the following three variants with approximately similar titers—PVY–Fel d 1, mPVY–Fel d 1 and PVY-SpyT/SpyC3-Fel d 1 (corresponding titers approximately 1:9000–10,000). The lowest IgG values were observed for PVY-SpyT/SpyC2-Fel d 1 (corresponding titer 1:3400), again as seen with rFel d 1, suggesting that this variant was not as immunogenic as others, possibly due to its inability to present antigens in a recognizable manner.

These findings strongly correlate with previously obtained data from avidity tests, confirming that cPVY–Fel d 1 produces Abs that are very specific to the VLP surface-exposed Fel d 1 antigen, whereas Abs from PVY-SpyT/SpyC2-Fel d 1 struggled to interact with the presented antigen, resulting in fewer nonspecific Abs. These results clearly demonstrate the effect of the chosen Fel d 1 vaccine design on the immune response and specificity of the elicited Abs.

## 4. Conclusions

In vaccine manufacturing, production platforms need to be simple. Here, we demonstrate that the Fel d 1 antigen can be introduced genetically into the PVY structure by direct and mosaic fusions as well as enzymatically by SpyTag/SpyCatcher–mediated antigen coupling to the VLP carrier. These processes are performed directly in *E. coli* cells, avoiding multiple separate processes, such as the production and purification of VLPs in one process, antigen production and purification in a second process and the subsequent third process—reaction between both components. We managed to successfully clone and express four PVY CP VLPs containing the genetically fused Fel d 1 antigen directly in *E. coli* cells, resulting in stable PVY-like filamentous VLP production. All obtained constructs were well expressed, soluble, and had Fel d 1 incorporation confirmed by WB and ELISA analysis using mAbs. Chemical coupling of Fel d 1 on surface-exposed lysines of PVY VLPs was also successful, allowing us to compare five different PVY–Fel d 1 vaccine variants in murine models.

A number of plant viruses have been used for new vaccine platform development, exploiting chemical, physical, genetic, and immunological aspects for the best possible outcome [1]. Immunology studies of VLPs as vaccine carriers demonstrate that antigens larger than 200 µm cannot freely enter the lymphatic system due to their time-consuming active uptake mechanism, resulting in fewer total IgG Abs than icosahedral particles ~30 nm in size [20,41,42].

It is known that the immunological features of VLPs depend on repetitive and particulate structures and induction of innate immunity through activation of pathogen-associated molecular pattern recognition receptors (PAMPs) [3]. A recent study by Pitek et al. [43] observed the interaction between VLPs of different shapes and surface chemistry and plasma proteins, leading to the conclusion that different surface charges attract protein coronas with different compositions. They proposed a mechanism in which nanoparticle–cell interactions are enhanced by additional interactions with the cell surface-mediated corona proteins, which may enhance cell receptor clustering and interactions to promote uptake [43]. As a result, differences in surface charge can significantly influence the Ab titers. The constructed PVY-derived vaccines are differently charged: (1) chemical coupling modifies the Lys residues and decreases the positive charges on the VLP surface; and (2) adding the positively charged SpyTag on the surface of PVY VLPs increases the overall positive charge. These changes may explain the observed differences in our experiments with Ab titers and avidity. Additionally, epitope spacing on the VLP surface can affect the immune response: a 5–10 nm distance between antigen molecules is considered to be optimal to induce the best Ab response [44]. In our experiments, we did not observe a correlation between the percentage of VLP-incorporated Fel d 1 antigens and the immune response, so additional studies are necessary to elucidate the influence of these factors on the quality of Abs.

Interestingly, all of the constructed vaccine variants elicited high Ab production against Fel d 1, the use of cPVY–Fel d 1 resulted in the strongest immune response and highest avidity Ab, suggesting that Fel d 1 antigen attachment on the VLP surface via chemical coupling may be the most effective way to obtain an efficient vaccine against Fel d 1. On the other hand, this result may be explained by the presence of small particles in the cPVY–Fel d 1 VLP sample observed in EM (Figure 5A), therefore such strong immune reactions were elicited because the VLP size was previously proven to be significant in this context [20]. Moreover, Abs against cPVY–Fel d 1 demonstrated the best recognition of nFel d 1, which indicates that Abs generated after immunization by this variant are strongly specific to nFel d 1.

IgG2a subclass Abs are most effective against virus infection in mouse models [39]. However, in our study, the majority of produced Abs were IgG1 subclasses for all variants demonstrating switching toward the T_h_2 response. Several authors suggest that isotype switching toward the IgG2a subclass is strongly dependent on TLR ligands such as ssRNAs [39,45] which are naturally packed into VLPs during *E. coli* expression [29]. Therefore, improving TLR stimulation by using T_h_1–inducing adjuvants or packed TLR9 stimulators such as CpGs [29,46,47] should be considered.

Although the size and shape of VLPs were confirmed to be important, these factors may not be the only critical parameters. Surprisingly, the obtained results reveal that filamentous PVY–Fel d 1 variants induce similar titers of total IgGs when compared with those of recently published icosahedral Qb-Fel d 1 vaccines [48] or even exceed the IgG levels elicited by VLPs from CMV containing the same Fel d 1 antigen [16].

Our study clearly demonstrates that the chosen expression platform can significantly influence the immune response, and several vaccine variants must be tested to find the best vaccine variant ensuring the highest and most specific immune response. PVY CP-derived VLPs are an efficient platform for comparing various antigen presentation systems that can help to evaluate different vaccine designs. Moreover, PVY VLPs might be used in the future for the development of the vaccine against cat allergy and other clinically significant vaccines.

## Data Availability

Not applicable.

## Supplementary Materials

The following are available online at https://www.mdpi.com/article/10.3390/vaccines10040485/s1, Supplement File: Plasmid maps and amino acid sequences. Figure S1: Description of plasmid pET-PVY-NG4S-Feld1; Figure S2: Description of plasmid pET-PVY-NG4S-Feld1-PVY; Figure S3: Description of plasmid pET-PVY-SpT-SpC2-Feld1; Figure S4: Description of plasmid pET-PVY-SpT-SpC2-Feld1.
