# Supplementary material for: Comparison of Bacterial Expression Systems Based on Potato Virus Y-like Particles for Vaccine Generation
Source: Vaccines (Basel). 2022 Mar 22;10(4):485. doi: 10.3390/vaccines10040485 (PMC9030781; doi:10.3390/vaccines10040485)
Supplement: Supplementary file 1 [file vaccines-10-00485-s001.zip › vaccines-1633981-supplementary.pptx]

## Slide 1
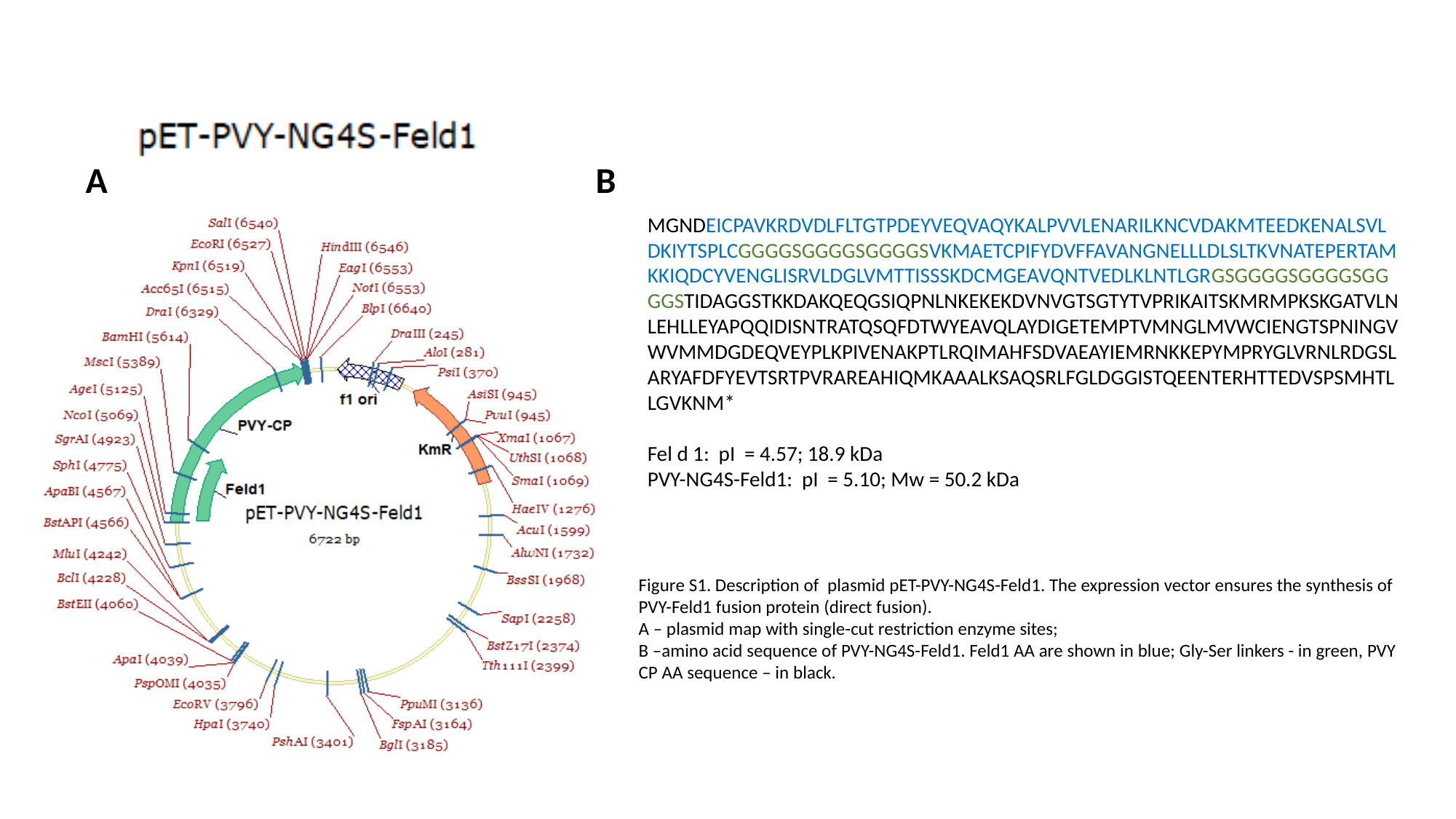

A
B
MgndeicpavkrdvdlfltgtpdeyveqvaqykalpvvlenarilkncvdakmteedkenalsvldkiytsplcggggsggggsggggsvkmaetcpifydvffavangnellldlsltkvnatepertamkkiqdcyvenglisrvldglvmttissskdcmgeavqntvedlklntlgrgsggggsggggsggggstidaggstkkdakqeqgsiqpnlnkekekdvnvgtsgtytvPrikaitskmrmpkskgatvlnlehlleyapqqidisntratqsqfdtwyeavqlaydigetemptvmnglmvwcieNgtspningvwvmmdgdeqveyplkpivenakptlrqimahfsdvaeayiemrnkkepympryglvrnlrdgslaryafdfyevtsrtpvrareahiqmkaaalksaqsrlfgldggistqeenterhttedvspsmhtllgvknm*
Fel d 1: pI = 4.57; 18.9 kDa
PVY-NG4S-Feld1: pI = 5.10; Mw = 50.2 kDa
Figure S1. Description of plasmid pET-PVY-NG4S-Feld1. The expression vector ensures the synthesis of PVY-Feld1 fusion protein (direct fusion).
A – plasmid map with single-cut restriction enzyme sites;
B –amino acid sequence of PVY-NG4S-Feld1. Feld1 AA are shown in blue; Gly-Ser linkers - in green, PVY CP AA sequence – in black.

## Slide 2
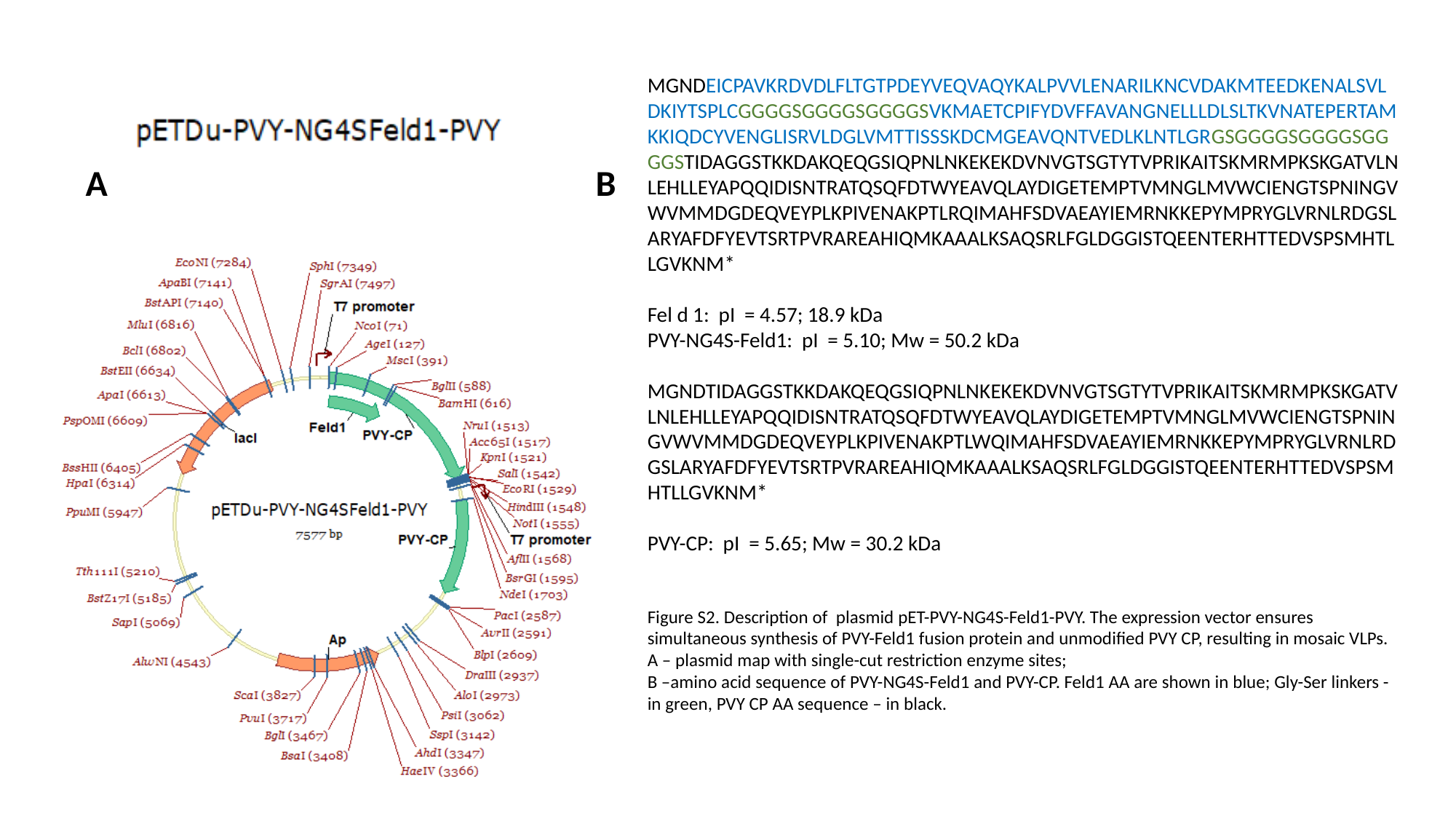

MgndeicpavkrdvdlfltgtpdeyveqvaqykalpvvlenarilkncvdakmteedkenalsvldkiytsplcggggsggggsggggsvkmaetcpifydvffavangnellldlsltkvnatepertamkkiqdcyvenglisrvldglvmttissskdcmgeavqntvedlklntlgrgsggggsggggsggggstidaggstkkdakqeqgsiqpnlnkekekdvnvgtsgtytvPrikaitskmrmpkskgatvlnlehlleyapqqidisntratqsqfdtwyeavqlaydigetemptvmnglmvwcieNgtspningvwvmmdgdeqveyplkpivenakptlrqimahfsdvaeayiemrnkkepympryglvrnlrdgslaryafdfyevtsrtpvrareahiqmkaaalksaqsrlfgldggistqeenterhttedvspsmhtllgvknm*
Fel d 1: pI = 4.57; 18.9 kDa
PVY-NG4S-Feld1: pI = 5.10; Mw = 50.2 kDa
mgndtidaggstkkdakqeqgsiqpnlnkekekdvnvgtsgtytvprikaitskmrmpkskgatvlnlehlleyapqqidisntratqsqfdtwyeavqlaydigetemptvmnglmvwciengtspningvwvmmdgdeqveyplkpivenakptlwqimahfsdvaeayiemrnkkepympryglvrnlrdgslaryafdfyevtsrtpvrareahiqmkaaalksaqsrlfgldggistqeenterhttedvspsmhtllgvknm*
PVY-CP: pI = 5.65; Mw = 30.2 kDa
A
B
Figure S2. Description of plasmid pET-PVY-NG4S-Feld1-PVY. The expression vector ensures simultaneous synthesis of PVY-Feld1 fusion protein and unmodified PVY CP, resulting in mosaic VLPs.
A – plasmid map with single-cut restriction enzyme sites;
B –amino acid sequence of PVY-NG4S-Feld1 and PVY-CP. Feld1 AA are shown in blue; Gly-Ser linkers - in green, PVY CP AA sequence – in black.

## Slide 3
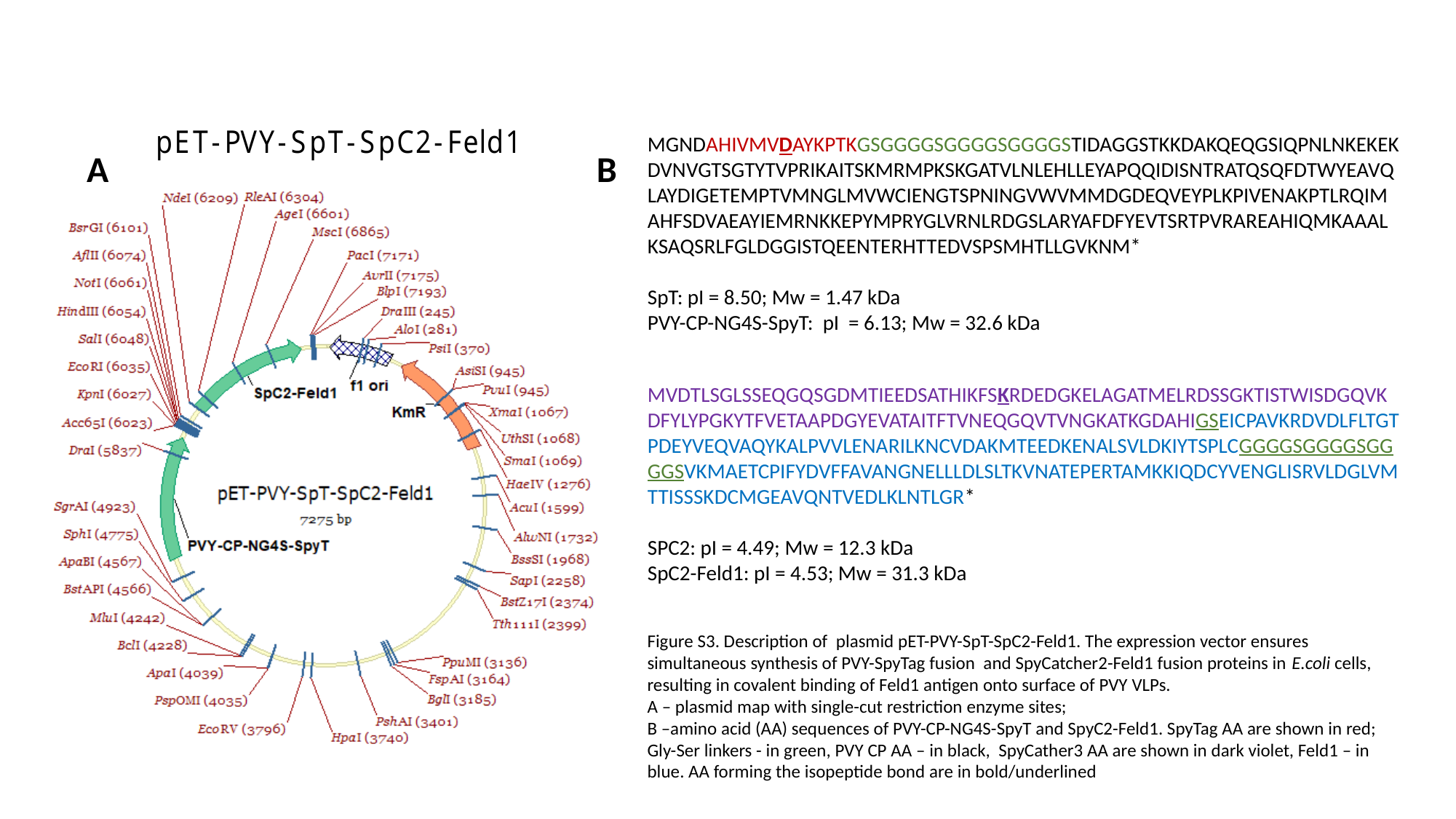

MgndahivmvdaykptkgsggggsggggsggggstidaggstkkdakqeqgsiqpnlnkekekdvnvgtsgtytvPrikaitskmrmpkskgatvlnlehlleyapqqidisntratqsqfdtwyeavqlaydigetemptvmnglmvwcieNgtspningvwvmmdgdeqveyplkpivenakptlrqimahfsdvaeayiemrnkkepympryglvrnlrdgslaryafdfyevtsrtpvrareahiqmkaaalksaqsrlfgldggistqeenterhttedvspsmhtllgvknm*
SpT: pI = 8.50; Mw = 1.47 kDa
PVY-CP-NG4S-SpyT: pI = 6.13; Mw = 32.6 kDa
A
B
mvdtlsglsseqgqsgdmtieedsathikfskrdedgkelagatmelrdssgktistwisdgqvkdfylypgkytfvetaapdgyevataitftvneqgqvtvngkatkgdahigseicpavkrdvdlfltgtpdeyveqvaqykalpvvlenarilkncvdakmteedkenalsvldkiytsplcggggsggggsggggsvkmaetcpifydvffavangnellldlsltkvnatepertamkkiqdcyvenglisrvldglvmttissskdcmgeavqntvedlklntlgr*
SpC2: pI = 4.49; Mw = 12.3 kDa
SpC2-Feld1: pI = 4.53; Mw = 31.3 kDa
Figure S3. Description of plasmid pET-PVY-SpT-SpC2-Feld1. The expression vector ensures simultaneous synthesis of PVY-SpyTag fusion and SpyCatcher2-Feld1 fusion proteins in E.coli cells, resulting in covalent binding of Feld1 antigen onto surface of PVY VLPs.
A – plasmid map with single-cut restriction enzyme sites;
B –amino acid (AA) sequences of PVY-CP-NG4S-SpyT and SpyC2-Feld1. SpyTag AA are shown in red; Gly-Ser linkers - in green, PVY CP AA – in black, SpyCather3 AA are shown in dark violet, Feld1 – in blue. AA forming the isopeptide bond are in bold/underlined

## Slide 4
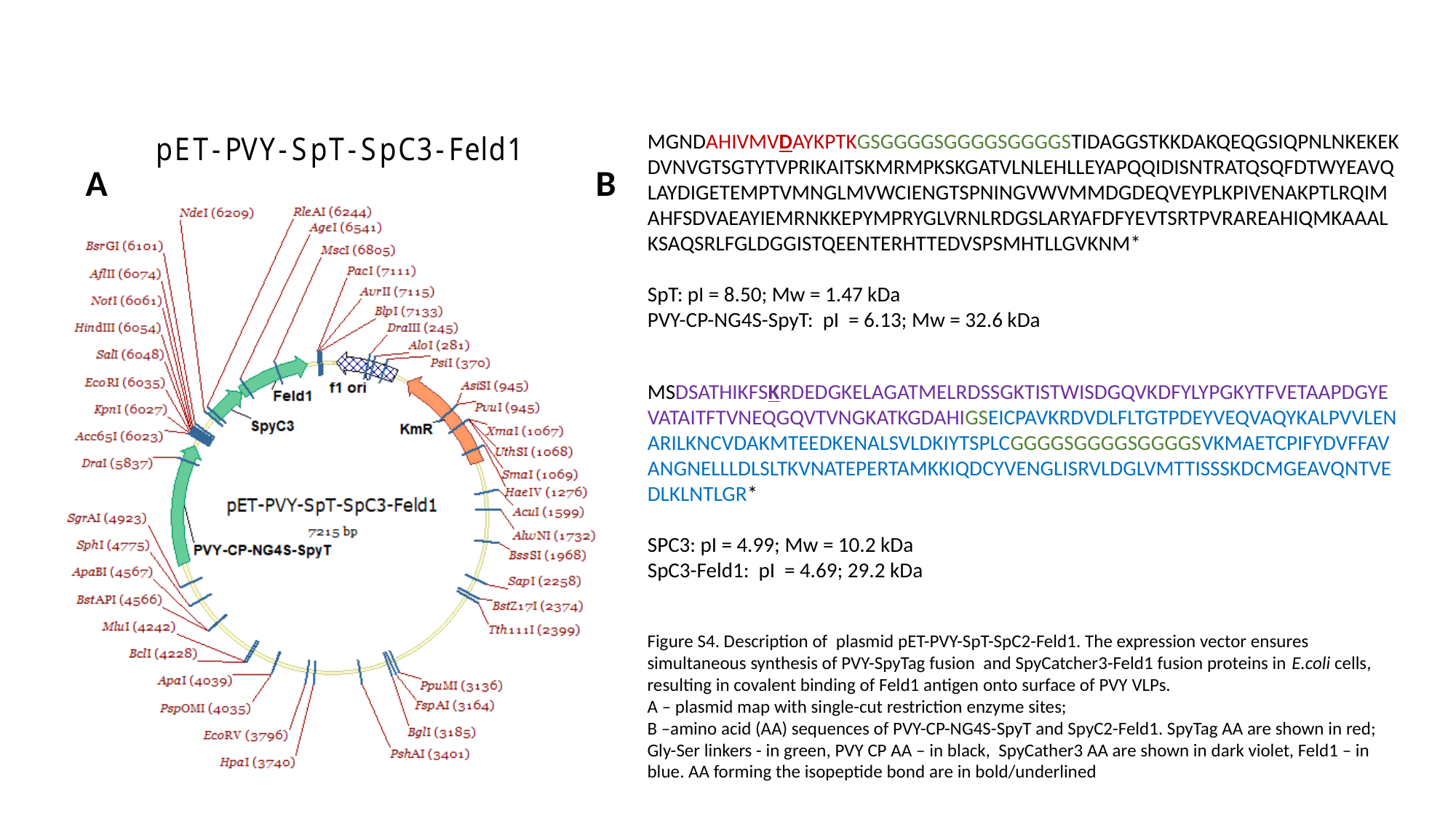

MgndahivmvdaykptkgsggggsggggsggggstidaggstkkdakqeqgsiqpnlnkekekdvnvgtsgtytvPrikaitskmrmpkskgatvlnlehlleyapqqidisntratqsqfdtwyeavqlaydigetemptvmnglmvwcieNgtspningvwvmmdgdeqveyplkpivenakptlrqimahfsdvaeayiemrnkkepympryglvrnlrdgslaryafdfyevtsrtpvrareahiqmkaaalksaqsrlfgldggistqeenterhttedvspsmhtllgvknm*
SpT: pI = 8.50; Mw = 1.47 kDa
PVY-CP-NG4S-SpyT: pI = 6.13; Mw = 32.6 kDa
A
B
mSdsathikfskrdedgkelagatmelrdssgktistwisdgqvkdfylypgkytfvetaapdgyevataitftvneqgqvtvngkatkgdahigseicpavkrdvdlfltgtpdeyveqvaqykalpvvlenarilkncvdakmteedkenalsvldkiytsplcggggsggggsggggsvkmaetcpifydvffavangnellldlsltkvnatepertamkkiqdcyvenglisrvldglvmttissskdcmgeavqntvedlklntlgr*
SpC3: pI = 4.99; Mw = 10.2 kDa
SpC3-Feld1: pI = 4.69; 29.2 kDa
Figure S4. Description of plasmid pET-PVY-SpT-SpC2-Feld1. The expression vector ensures simultaneous synthesis of PVY-SpyTag fusion and SpyCatcher3-Feld1 fusion proteins in E.coli cells, resulting in covalent binding of Feld1 antigen onto surface of PVY VLPs.
A – plasmid map with single-cut restriction enzyme sites;
B –amino acid (AA) sequences of PVY-CP-NG4S-SpyT and SpyC2-Feld1. SpyTag AA are shown in red; Gly-Ser linkers - in green, PVY CP AA – in black, SpyCather3 AA are shown in dark violet, Feld1 – in blue. AA forming the isopeptide bond are in bold/underlined
